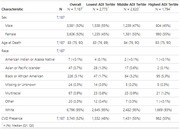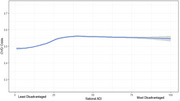# Association of Neighborhood‐Level Disadvantage with Cerebrovascular Disease Neuropathology in a National Sample of Brain Donors

**DOI:** 10.1002/alz70855_100558

**Published:** 2025-12-23

**Authors:** Grace C George, Sarah A. Keller, W. Ryan Powell, William R. Buckingham, Barbara B. Bendlin, Erin L. Abner, Liana G. Apostolova, Carmen Carrion, Thomas K Karikari, Oanh L. Meyer, Robert A. Rissman, Patricia Rodriguez Espinoza, Rachel A. Whitmer, Amy J.H. Kind, The Neighborhoods Study Scientific Team

**Affiliations:** ^1^ Center for Health Disparities Research, University of Wisconsin School of Medicine and Public Health, Madison, WI, USA; ^2^ Department of Epidemiology and Environmental Health, University of Kentucky, Lexington, KY, USA; ^3^ Indiana University School of Medicine, Indianapolis, IN, USA; ^4^ Yale University, New Haven, CT, USA; ^5^ University of Pittsburgh, Pittsburgh, PA, USA; ^6^ University of California, Davis School of Medicine, Sacramento, CA, USA; ^7^ Alzheimer's Therapeutic Research Institute, Keck School of Medicine, University of Southern California, San Diego, CA, USA; ^8^ Stanford University School of Medicine, Palo Alto, CA, USA; ^9^ University of California Davis, Davis, CA, USA

## Abstract

**Background:**

Alzheimer's Disease and related dementias (ADRD) are complex disorders with increased risk in populations experiencing structural inequities, including those who live in disadvantaged neighborhoods as indexed by the Area Deprivation Index (ADI). Increasingly, evidence suggests multi‐etiology dementia neuropathology in these populations. Cerebrovascular Disease (CVD) is common in multi‐etiology dementia neuropathology. Currently, little work has investigated ADI's relationship with CVD. Through the Neighborhoods Study, a national study leveraging 21 Alzheimer's Disease Research Center (ADRC) brain banks to evaluate the adverse social exposomes relationship to neuropathology, we sought to understand weather high deprivation predicts CVD in this national sample of brain bank donors.

**Method:**

21 ADRC brain banks and their brain donor participants were eligible (*N* = 7187) as part of the Neighborhoods Study. Addresses at death were linked to national ADI percentiles corresponding to year of death, organized into categorical statistical tertiles (1‐20 (*N* = 2773), 21‐50 (*N* = 2620), 51‐100 (*N* = 1794). Outcomes included presence of a selection of CVD pathologic changes and lesions (infarcts, hemorrhages, microinfarcts, and white matter rarefaction) as noted within NACC neuropathology data, as per method put forward by Godrich et al., 2022. We used a logistic regression adjusting for sex, age at death, and APOE carrier status.

**Result:**

We found that ∼25% of donors lived in high deprivation contexts, 52% had at least one incidence of CVD, and the cohort identified as <1% American Indian or Native Alaskan, <1 % Asian or Pacific Islander, ∼1% Multiracial, ∼3% Black, and ∼95% White. We found that increased ADI predicted significantly increased odds of CVD neuropathology in our cohort (OR = 1.16, *p* <.001). Further, we found that the low deprivation had significantly fewer donors with CVD (48%) compared to the middle (54.6% CVD, OR = 1.30, *p* < .001) and highest tertiles (54.7% CVD, OR=1.34, *p* <.001); however, there was no significant difference between the middle and highest tertiles (*p* > .05).

**Conclusion:**

We found that more disadvantaged neighborhoods predict CVD neuropathology in a nationwide brain donor cohort in the Neighborhoods Study. Further work will investigate the mechanisms that might contribute to the prevalence of overlapping multi‐etiology neuropathology in persons residing in highly disadvantaged neighborhoods.